# County-Level Variation in Prevalence of Multiple Chronic Conditions Among Medicare Beneficiaries, 2012

**DOI:** 10.5888/pcd12.140442

**Published:** 2015-01-22

**Authors:** Kimberly A. Lochner, Carla M. Shoff

**Affiliations:** Author Affiliation: Carla M. Shoff, PhD, Centers for Medicare & Medicaid Services, Baltimore, Maryland.

**Figure:**
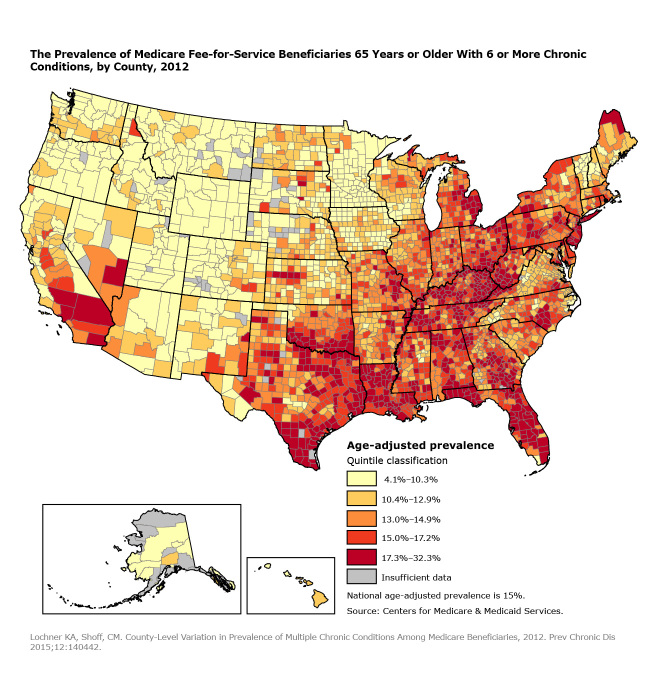
The map illustrates the geographic variation across counties and shows that counties with the highest prevalence of Medicare beneficiaries with 6 or more chronic conditions are located predominantly in southern states (eg, Texas, Florida, Kentucky) and northeastern states (eg, New York, Pennsylvania). Counties with the lowest prevalence are found mostly in western states (eg, Oregon, Montana, Wyoming).

## Background

Preventing chronic conditions and controlling costs associated with the care for people with chronic conditions are public health and health care priorities. The number of chronic conditions increase with age: more than two-thirds of Medicare beneficiaries 65 years or older have 2 or more chronic conditions, and more than 15% have 6 or more (1,2). People with multiple chronic conditions use more health care services than people who do not have them, and they account for a disproportionate share of health care spending (2,3). The prevalence of multiple chronic conditions varies substantially by state (4); more granular geographic information on multiple chronic conditions can provide a better understanding of the burden of chronic conditions and the implications for local public health programs and resources. The objective of this geographic information system (GIS) analysis was to describe county-level prevalence patterns of Medicare beneficiaries with 6 or more chronic conditions.

## Methods

We estimated the prevalence of beneficiaries with 6 or more chronic conditions by county using Centers for Medicare & Medicaid Services (CMS) administrative enrollment and claims data for 100% of Medicare beneficiaries enrolled in the fee-for-service program in 2012; we excluded Medicare beneficiaries enrolled in Medicare Advantage and those enrolled in Medicare Part A only or Part B only. We also excluded Medicare beneficiaries under the age of 65 who are entitled to Medicare because of a disability. These exclusions resulted in 27.9 million fee-for-service beneficiaries 65 years or older; we refer to them as “aged” beneficiaries based on their eligibility for Medicare. Prevalence estimates of multiple chronic conditions may vary from one source of data to another, and estimates are further influenced by the number and type of conditions included in the analysis. We chose 6 chronic conditions from a set of 17 chronic conditions previously defined (1). A Medicare beneficiary is considered to have a chronic condition if a Medicare claim indicates that the beneficiary received a service or treatment for the condition. The study population and data sources are described in detail elsewhere (2,5).

We calculated prevalence estimates by dividing the number of beneficiaries with 6 or more of the 17 chronic conditions by the total number of beneficiaries in our fee-for-service population, expressed as a percentage. We directly age-adjusted all prevalence estimates to the 2000 US standard population aged 65 or older. An age-adjusted rate is a weighted average of age-specific rates calculated on the basis of the proportion of people in the corresponding age groups of a standard population. Age adjustment allows for a comparison of rates between counties that have different age distributions (6). We suppressed data on counties with fewer than 100 beneficiaries or fewer than 20 beneficiaries with 6 or more chronic conditions (2.2% of US counties). We categorized prevalence estimates into quintiles. We did not determine significant differences among estimates. 

## Main Findings

In 2012, 15% of aged Medicare beneficiaries had 6 or more chronic conditions. Prevalence varied geographically by county; counties in the lowest quintile had prevalence estimates of 10.3% or lower, and those in the highest quintile had prevalence estimates of 17.3% or higher. Counties in the highest quintile had prevalence estimates that were 1.2 times higher than the national average of 15%. Eighty-seven counties had estimates at least 1.5 times higher than the national average; 3 counties had prevalence estimates at least twice the national average. Counties in the Northeast and Southeast generally had a higher prevalence of aged beneficiaries with 6 or more chronic conditions than the national average, whereas counties with prevalence estimates below the national average were predominantly in the western states of Oregon, Montana, and Wyoming. 

## Action

State health departments and other local health partners play key roles in surveillance, program planning, and resource allocation to prevent and manage chronic diseases among their populations. This map illustrates the county-level variability for 1 indicator of multiple chronic conditions — aged Medicare beneficiaries with 6 or more chronic conditions — and the importance of having granular geographic data on chronic conditions. CMS has made data resources on chronic conditions among Medicare beneficiaries publicly available. These resources include data tables, maps, and interactive dashboards at the state and county levels (2), which can fill information gaps and be used to better inform decision making for policies and to target program and services. We hope that the geographic patterns shown by our map helps to stimulate further investigation of the prevalence of multiple chronic conditions in the United States.
